# Supplementary material for: Photoaging and Sequential Function Reversal with Cellular-Resolution Optical Coherence Tomography in a Nude Mice Model
Source: Int J Mol Sci. 2022 Jun 23;23(13):7009. doi: 10.3390/ijms23137009 (PMC9266384; doi:10.3390/ijms23137009)
Supplement: Supplementary file 1 [file ijms-23-07009-s001.zip › ijms-1757786-supplementary.pdf]

International Journal of
Molecular Sciences

Supplementary Material

Table S1. The advantages of biopsy and in vivo cellular resolution full-field optical coherence tomography (FFOCT).

**Biopsy**	**In vivo cellular resolution FFOCT**
Single point<br>Improper sampling	Non-invasive and repetitive examination
Scar and infection risk； interpretation interference post biopsy related inflammation	Touchless
Limited numbers of images	Huge number of images
Complex deep learning process for AI training	AI-assisted recognition and quantification
Details of cytoplasmic, nuclear, and extracellular matrix, inflammatory cells types	Keratinocytes nuclei shape, size, collagen amount, vessels.<br>Cannot differentiate inflammatory cells types
Access for gene survey available	No access for gene information

AI: artificial intelligence; FFOCT: full-field optical coherence tomography.

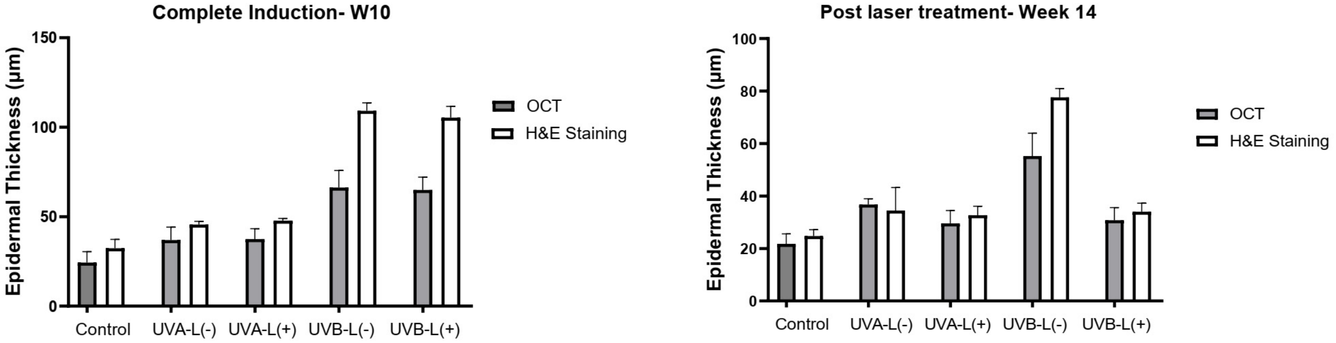

Figure S1. Compare epidermal thickness measured by OCT/ Hematoxylin and Eosin (H&E) staining.

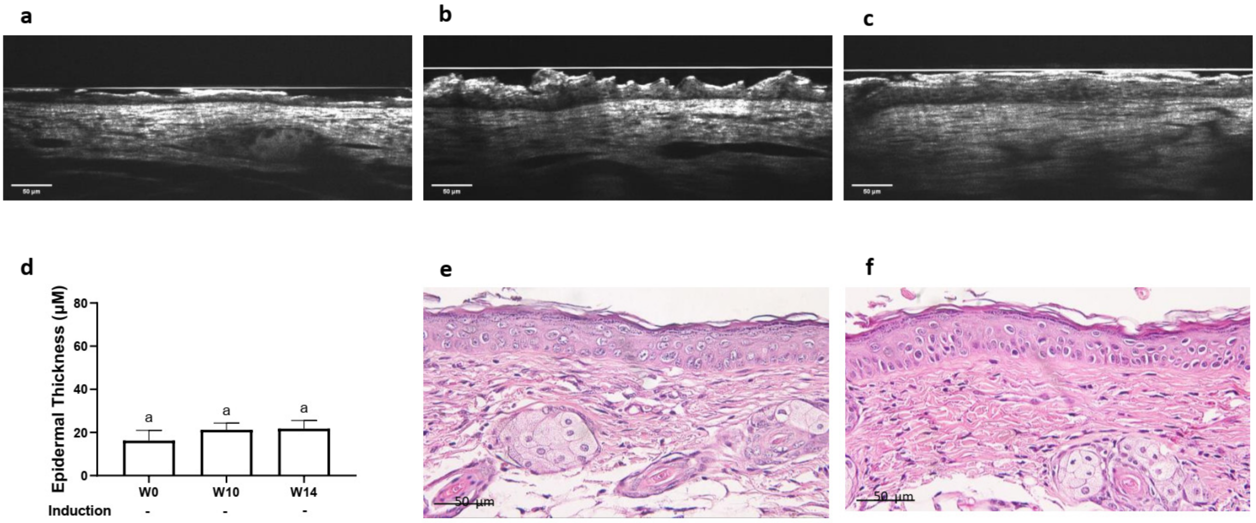

Figure S2. The control group mice did not have obvious skin changes with time (W0, W10, W14 from left to right).

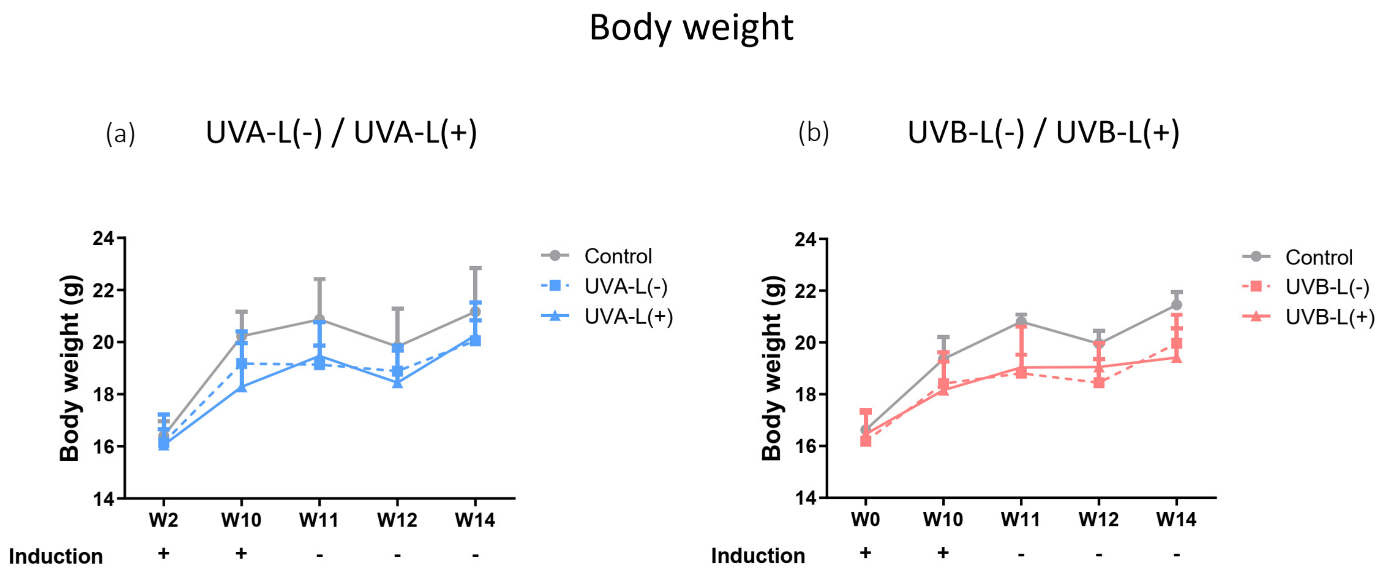

Figure S3. Body weight of nude mice in chronic (**a**) UVA-irradiation (**b**) UVB-irradiation over 14 weeks. At the end of the experiment, no considerable differences were observed in the body weights of the mice in all three groups.

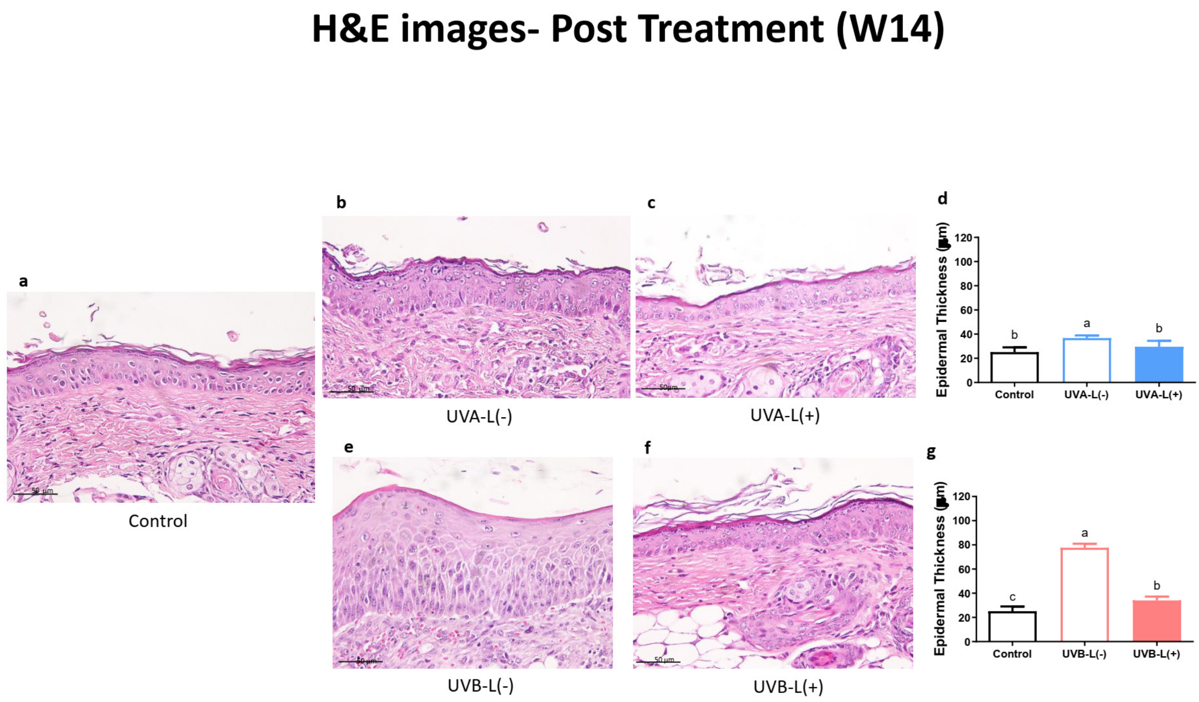

Figure S4. H&E staining images at the end of experiment (W14) of (a) control group, (b) UVA-L(-) group, (c) UVA-L(+) group, (e) UVB-L(-) group, (f) UVB-L(+) group. UVA-L(-) and UVB-L(-) group had 4 weeks of spontaneous recovery after UV insult halted while UVA-L(+) and UVB-L(+) group immediately received laser for 3 days followed by accelerated recovery. Note the intercellular edema was still obvious in UVB group (e), while UVB-L(+) group had regained epidermal polarity (f). Laser accelerated epidermal thickness recovery in both UVA-L(+) and UVB-L(+) group (d,g). Different letters indicate statistically significant differences at $p < 0.01$.

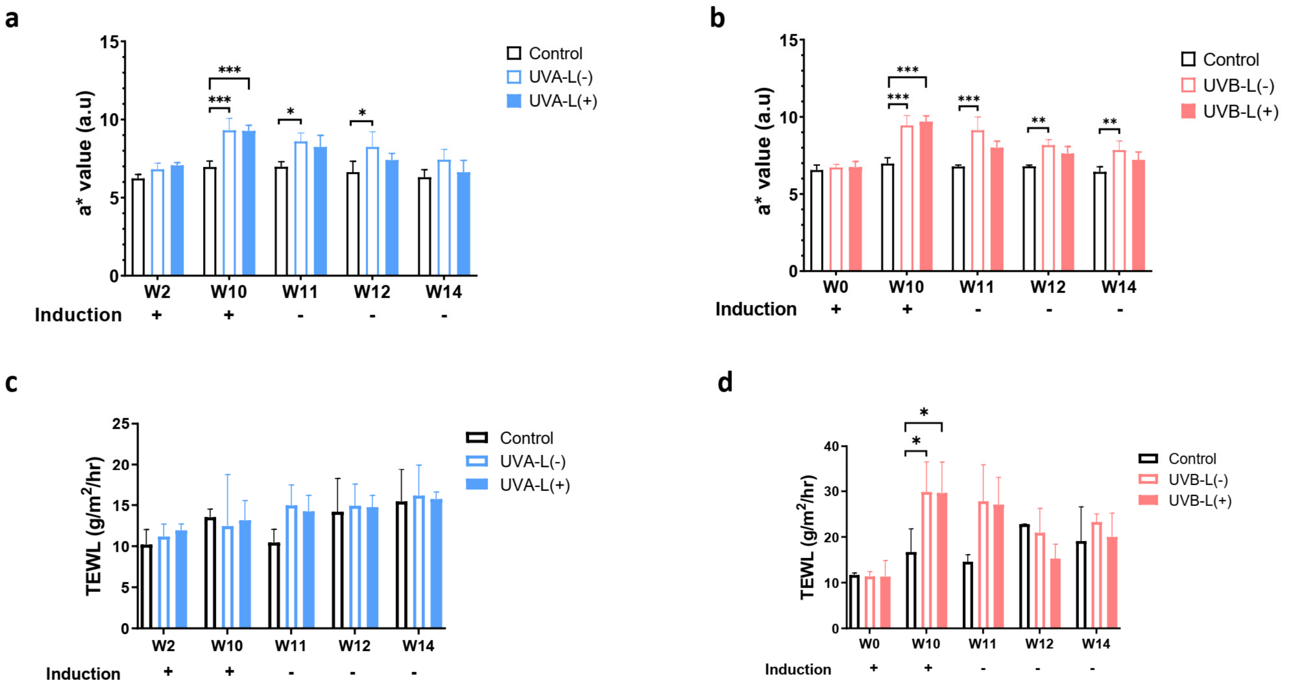

Figure S5. Effect of 755 nm picosecond laser on a* value (erythema) and transepidermal water loss (TEWL) (a,c) UVA-irradiation (b,d) UVB-irradiation nude mice. W2: UVA baseline; W10: complete induction *, p < 0.05; ***, p < 0.001: Significant difference versus control group.

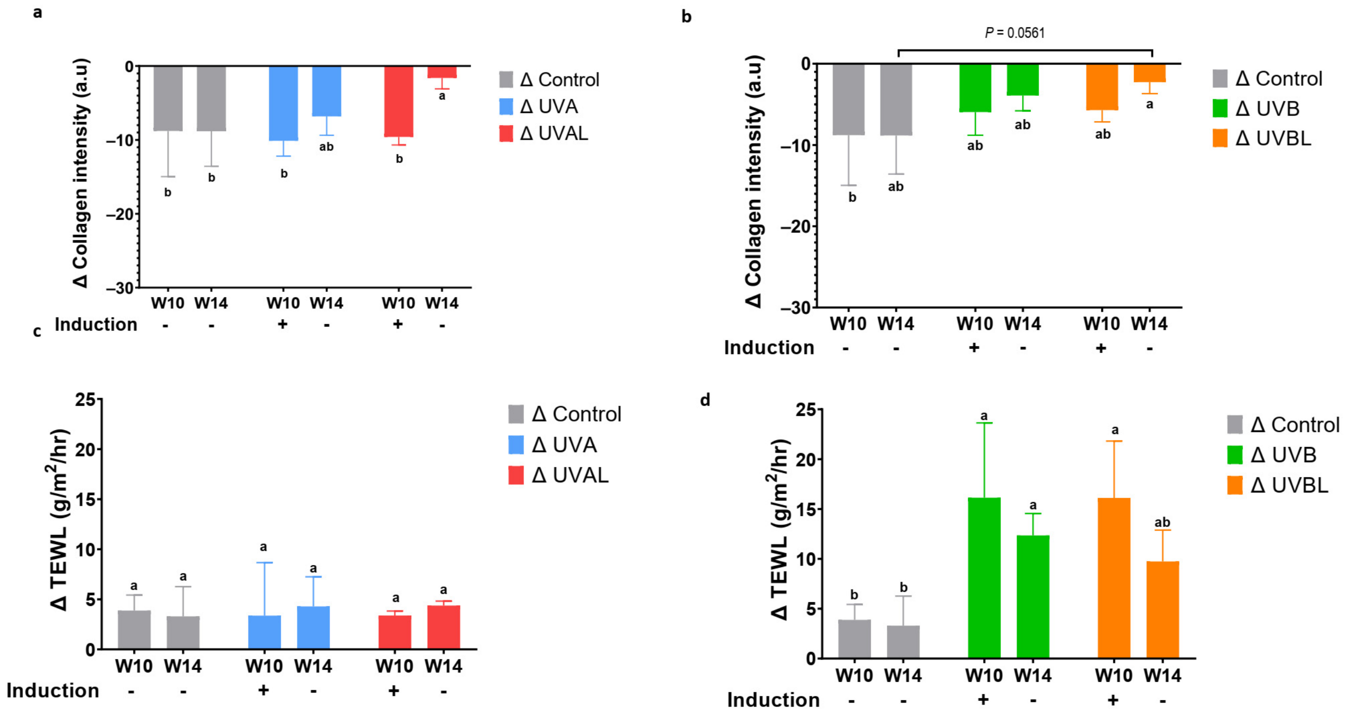

Figure S6. ΔTEWL and Δcollagen intensity (difference between W10/W14 and baseline) after induction(W10) and 4 weeks after induction (W14). Different letters indicate statistically significant differences at $p< 0.05$.
